# Bone marrow stroma-derived PGE_2_ protects BCP-ALL cells from DNA damage-induced p53 accumulation and cell death

**DOI:** 10.1186/s12943-014-0278-9

**Published:** 2015-01-27

**Authors:** Elin Hallan Naderi, Seham Skah, Hege Ugland, Ola Myklebost, Dagny Lise Sandnes, Maria Lyngaas Torgersen, Dag Josefsen, Ellen Ruud, Soheil Naderi, Heidi Kiil Blomhoff

**Affiliations:** Department of Biochemistry, Institute of Basic Medical Sciences, University of Oslo, PO Box 1112, Blindern, N-0317 Oslo Norway; Department of Tumour Biology, Institute of Cancer Research, Oslo University Hospital, Oslo, Norway; Department of Pharmacology, Institute of Clinical Medicine, University of Oslo, Oslo, Norway; Department of Cellular Therapy, Oslo University Hospital, Oslo, Norway; Department of Paediatrics, Oslo University Hospital, Oslo, Norway; Present address: Department of Medical Genetics, Oslo University Hospital, Oslo, Norway

**Keywords:** Acute lymphoblastic leukaemia, Tumour stroma, DNA damage, p53, cAMP, PGE_2_

## Abstract

**Background:**

B cell precursor acute lymphoblastic leukaemia (BCP-ALL) is the most common paediatric cancer. BCP-ALL blasts typically retain wild type p53, and are therefore assumed to rely on indirect measures to suppress transformation-induced p53 activity. We have recently demonstrated that the second messenger cyclic adenosine monophosphate (cAMP) through activation of protein kinase A (PKA) has the ability to inhibit DNA damage-induced p53 accumulation and thereby promote survival of the leukaemic blasts.

Development of BCP-ALL in the bone marrow (BM) is supported by resident BM-derived mesenchymal stromal cells (MSCs). MSCs are known to produce prostaglandin E_2_ (PGE_2_) which upon binding to its receptors is able to elicit a cAMP response in target cells. We hypothesized that PGE_2_ produced by stromal cells in the BM microenvironment could stimulate cAMP production and PKA activation in BCP-ALL cells, thereby suppressing p53 accumulation and promoting survival of the malignant cells.

**Methods:**

Primary BCP-ALL cells isolated from BM aspirates at diagnosis were cocultivated with BM-derived MSCs, and effects on DNA damage-induced p53 accumulation and cell death were monitored by SDS-PAGE/immunoblotting and flow cytometry-based methods, respectively. Effects of intervention of signalling along the PGE_2_-cAMP-PKA axis were assessed by inhibition of PGE_2_ production or PKA activity. Statistical significance was tested by Wilcoxon signed-rank test or paired samples *t* test.

**Results:**

We demonstrate that BM-derived MSCs produce PGE_2_ and protect primary BCP-ALL cells from p53 accumulation and apoptotic cell death. The MSC-mediated protection of DNA damage-mediated cell death is reversible upon inhibition of PGE_2_ synthesis or PKA activity. Furthermore our results indicate differences in the sensitivity to variations in p53 levels between common cytogenetic subgroups of BCP-ALL.

**Conclusions:**

Our findings support our hypothesis that BM-derived PGE_2_, through activation of cAMP-PKA signalling in BCP-ALL blasts, can inhibit the tumour suppressive activity of wild type p53, thereby promoting leukaemogenesis and protecting against therapy-induced leukaemic cell death. These novel findings identify the PGE_2_-cAMP-PKA signalling pathway as a possible target for pharmacological intervention with potential relevance for treatment of BCP-ALL.

## Background

B cell precursor acute lymphoblastic leukaemia (BCP-ALL) is a malignant neoplasm that occurs in both children and adults, with approximately 1 in 2000 developing the disease during childhood [1]. BCP-ALL is treated with multimodal chemotherapy, and the overall survival is approaching 90% [2,3]. In spite of major improvements in treatment outcome over the last decades, there are major challenges remaining. High risk subgroups defined by various cytogenetic, biochemical, and clinical criteria have inferior outcomes [1]. Relapses also occur in children with standard risk features, accounting for more than a third of the total BCP-ALL-associated death toll [2]. Furthermore, it is well-documented that survivors experience a wide range of serious long-term treatment-associated side effects such as secondary cancers, chronic organ damage, cognitive, and psychosocial difficulties [4-7], emphasizing the need for further improvement of treatment strategies.

Paediatric BCP-ALLs generally retain wild type p53 [8], and are thus forced to suppress p53 activity through indirect measures. We have previously demonstrated that elevated levels of cyclic adenosine monophosphate (cAMP) can suppress DNA damage-induced p53 accumulation by promoting the interaction between p53 and its negative regulator HDM2, thereby attenuating the resulting cell death in BCP-ALL blasts [9-11]. cAMP is an important physiological signal transducer in lymphocytes [12-14], and it is generated by adenylate cyclase (AC) upon ligand binding to a subgroup of G protein-coupled receptors (GPCRs) activating the stimulatory alpha subunit G_αs_. This, together with our observation that cAMP attenuates the expression of p53 led us to postulate that elevated cAMP levels might function as a tumour promoting mechanism in development of BCP-ALL. The basal levels of intracellular cAMP in isolated BCP-ALLs are comparable to those of normal BCPs [11], and we therefore considered the possibility that BCP-ALLs could produce high levels of cAMP in response to certain physiological extracellular signals.

It has become increasingly clear that the biology of cancer cannot be understood simply by elucidating the cell-autonomous properties of the transformed cell population. Cancer cells live in a complex interrelation with non-transformed cells and extracellular matrix constituting the tumour microenvironment (ME), which play decisive roles in the development of malignant disease [15]. Much of the research into the role of tumour ME has been conducted in carcinomas in which diverse cell types such as endothelial cells, pericytes, immune inflammatory cells, and cancer-associated fibroblasts have been recognized to play crucial roles in malignant progression and the metastatic process [15]. Importantly, the bone marrow (BM) has increasingly been implicated as a key source of tumour-associated stromal cells, and evidence suggests that BM mesenchymal stem and progenitor cells are recruited to tumour sites where they can trans-differentiate into cells participating in tumour development, immunomodulation, and metastatic niche formation [16]. Such MSCs can even be transformed by inflammation to become neoplastic carcinoma cells [17].

Being an inherent malignant disease of the bone marrow, ALL blasts have immediate access to BM mesenchymal stromal cells (MSCs). In the BM, distinct niches are thought to exist, providing signals necessary to maintain and regulate haematopoiesis. Early lymphoid progenitors reside in a niche that is spatially and cellularly distinct from the haematopoietic stem cell niche [18]. BM-MSCs have been shown to support ALL blast engraftment and survival through a variety of factors mediated by cell-cell contact [19,20], or secretion of paracrine signalling molecules [21]. MSCs have also been reported to promote leukaemia development more indirectly through local immune suppression [22].

BM-MSCs are known to secrete prostaglandin E_2_ (PGE_2_) [23], and ALL blasts have been demonstrated to express functional EP2 receptors, one of the AC-activating subclasses of PGE_2_ receptors [24,25]. The role of tumour stroma-derived PGE_2_ has been extensively studied in solid tumours [26], however, little is known about its potential role in leukaemia physiology. Having recently shown that PGE_2_ can suppress the p53 levels and cell death when added to cell cultures of BCP-ALL cells [11], we have now explored the possible role of BM stroma-derived PGE_2_ on BCP-ALL p53 levels and cell survival. In agreement with our hypothesis, our results suggest that bone marrow microenvironment (BMME)-secreted PGE_2_, through its inhibitory effects on p53 accumulation and cell death, could contribute to the protective effect of the BMME towards BCP-ALL cells. Furthermore, our results identify BMME-derived PGE_2_ signalling as a possible target in treatment of BCP-ALL.

## Results

### Cocultivation with MSC protects primary BCP-ALL cells from cell death

**Figure 1:**
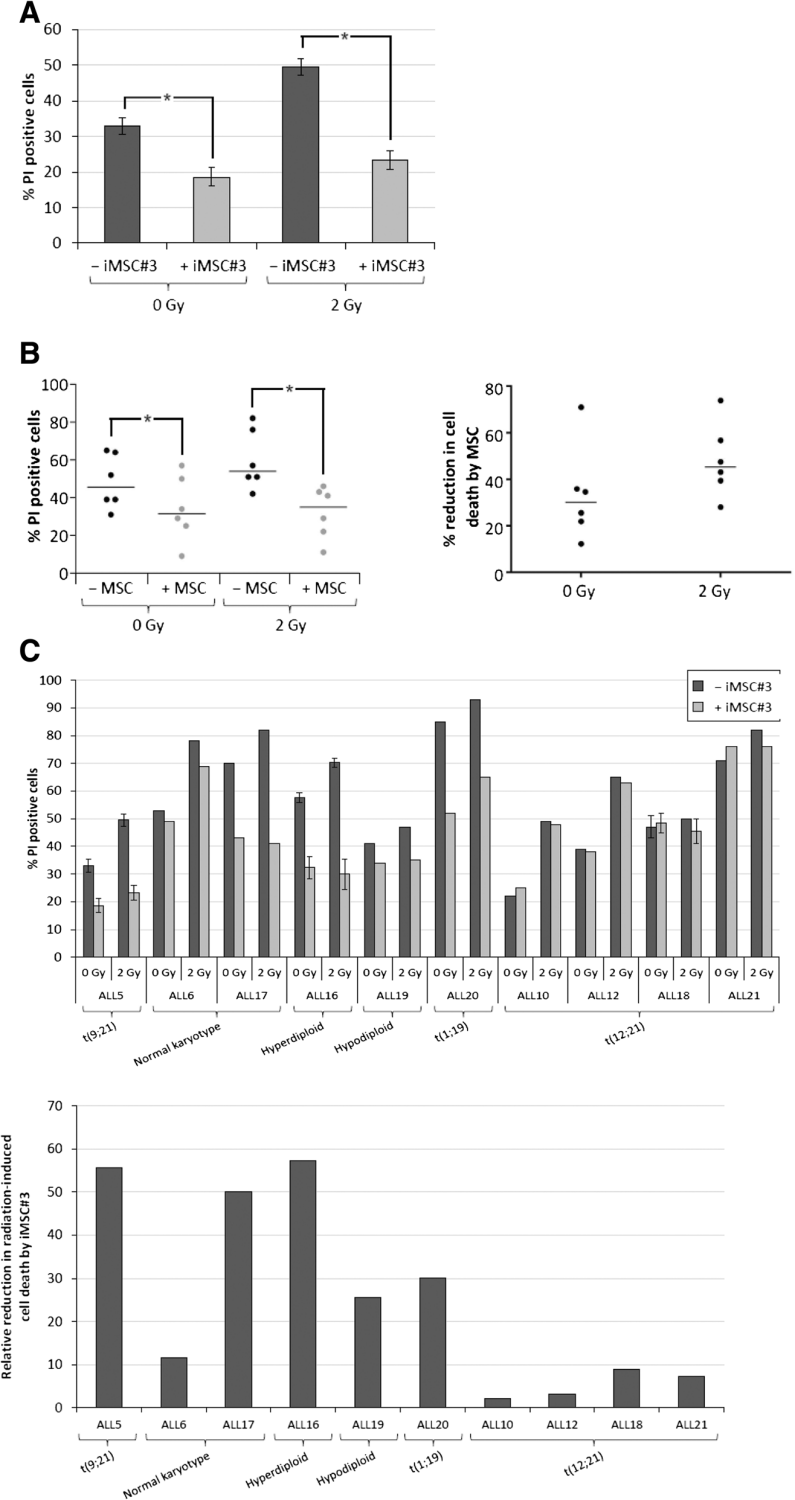
**MSC coculture protects primary BCP-ALL cells from cell death. (A)** Isolated BCP-ALL blasts from ALL5 were cultured in the absence or presence of a confluent layer of iMSC #3. After 2 h, the blasts were briefly removed from the coculture and irradiated with 2 Gy of IR. The cells were then reintroduced to the coculture and harvested after 20 h for examination of cell death in the CD19^+^ cell fraction by CD19-FITC/PI co-staining as described in Materials and Methods. The histogram shows mean values of 10 independent experiments, with error bars indicating SEM values. *P < 0.0001 by paired samples *t* test. **(B)** Isolated BCP-ALL blasts from ALL5 (n = 3) and ALL16 (n = 3) cultured in the absence or presence of a confluent layer of primary BM-MSC were treated and analysed as described in the legend to Figure 1A. The left panel shows absolute cell death values for each experiment with bars representing median values. *P < 0.05 by Wilcoxon matched pairs signed rank test. The right panel shows relative reduction in cell death mediated by MSC coculturing for each BCP-ALL sample at 0 Gy and 2 Gy of irradiation, respectively. The horizontal bars represent median values. **(C)** Isolated BCP-ALL blasts from 9 different patient samples were treated and analysed as described in Figure 1A, and grouped according to cytogenetic sub-classification. The upper panel shows absolute cell death values after 0 and 2 Gy of irradiation (ALL5: n = 10, error bars represent SEM values; ALL16: n = 3, error bars represent SEM values; ALL18: n = 2, error bars represent range of values; ALL6/10/12/17/19/20: values represent results from single experiments). The lower panel shows the relative reduction of cell death imposed by iMSC#3 on irradiated BCP-ALL blasts.

Previous work from our group demonstrated that PGE_2_, a naturally occurring eicosanoid secreted by the BMME, could replicate the effects of augmented cAMP levels to inhibit basal and DNA damage-induced p53 levels and cell death in primary BCP-ALL blasts [9,11]. We therefore hypothesized that PGE_2_ secreted by the BMME, through inhibitory effects on p53 accumulation and cell death, could contribute to the protective effect of the BMME towards BCP-ALL cells. To investigate this possibility further, we first needed to establish an *in vitro* model of BM protection of primary BCP-ALL cells. To this end, BCP-ALL blasts from ALL5 were cocultured on a confluent layer of the BM-derived MSC cell line iMSC#3. After 2 hours of coculture, the blasts were briefly removed and irradiated with 2 Gy of ionising radiation (IR). The cells were then reintroduced to the coculture and harvested after 20 hours for examination of cell death by propidium iodide (PI) staining and FACS analysis of the CD19^+^ cell fraction. The choice of IR as model system for inducing DNA damage has previously been discussed [11], and we have demonstrated similar effects of cAMP signalling on DNA-damaging cytostatic drugs such as anthracyclins, cyclophosphamide, and cisplatin [9]. As shown in Figure 1A, iMSC#3 in coculture significantly protected the leukaemic blasts against both spontaneous and IR-induced cell death.

To ascertain that the protective effect of iMSC#3 was not restricted to this cell line, primary MSCs were isolated and cocultured with BCP-ALL blasts from ALL5 and ALL16 under the same conditions as in Figure 1A. Similar to iMSC#3, the primary MSC layers provided a statistically significant protection against cell death with median reduction of IR-induced cell death of 45% (range 28%-74%) (Figure 1B). To examine the generality of this finding, isolated leukaemic blasts from nine different patients were subjected to 2 Gy of IR in the presence or absence of iMSC#3 as described above, and the resulting cell death was measured. As can be seen from the upper panel of Figure 1C, all patient samples displayed protection from cell death upon coculture with stromal cells. However, the relative degree of protection varied between the samples (Figure 1C, lower panel). Interestingly, t(12;21)-positive samples displayed a very weak protection against DNA damage-induced cell death, correlating with the inability of cAMP-elevating compounds to enhance the survival of ALL-blasts from patients with this particular translocation [11].

### MSC coculture inhibits DNA damage-induced p53 accumulation in primary BCP-ALL cells

**Figure 2:**
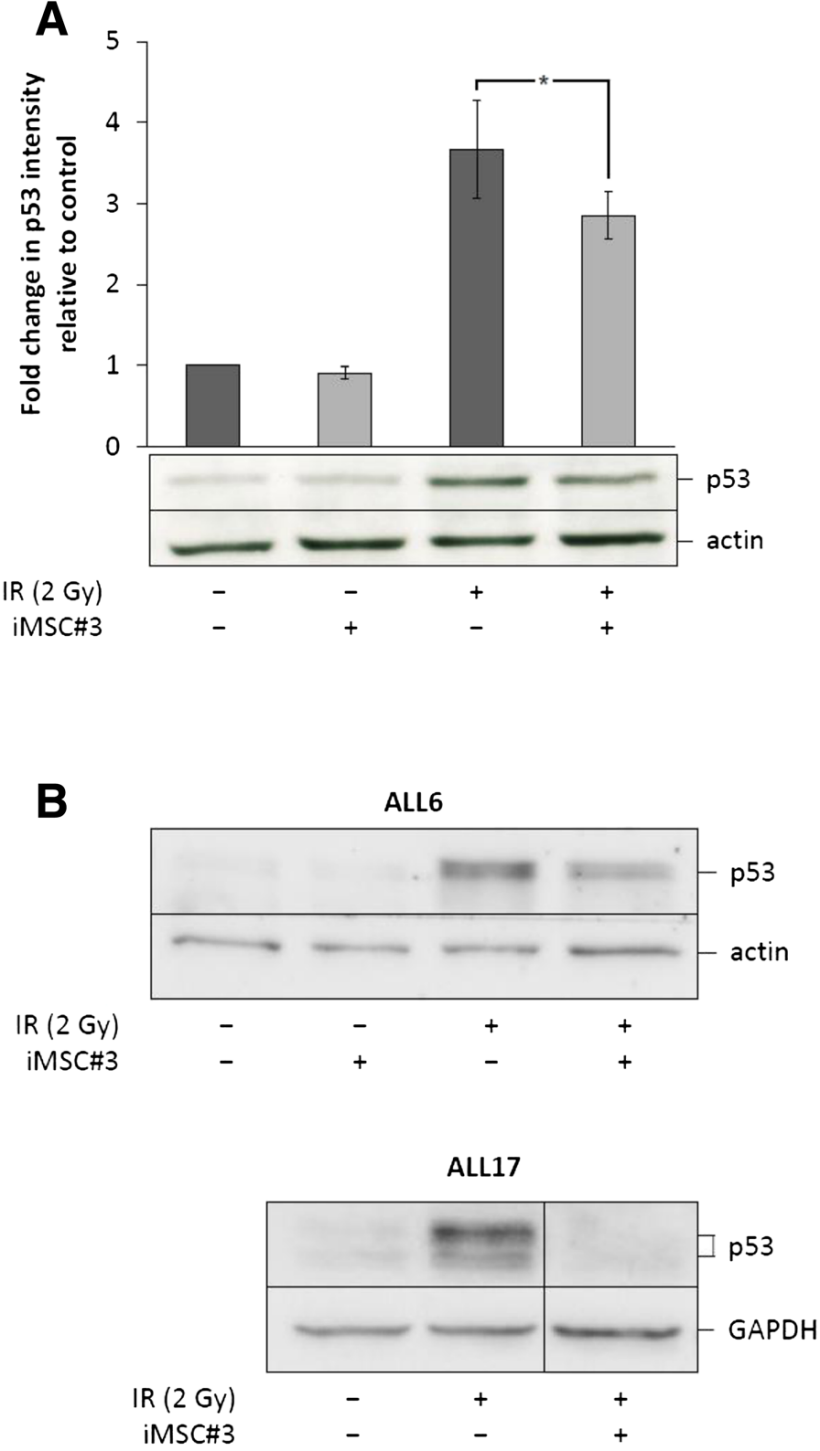
**MSC coculture inhibits DNA damage-induced p53 accumulation in primary BCP-ALL cells. (A)** BCP-ALL blasts from ALL5 were cultured in the absence or presence of a confluent layer of iMSC#3 and irradiated as described in Figure 1A. 2 h after IR, BCP-ALL blasts were harvested, lysed and subjected to immunoblot analysis with antibodies against p53 and actin. The immunoblot shows one representative experiment of four. Densitometric analysis of immunoblots was performed as described in Materials and Methods. The histogram shows mean values with error bars indicating SEM values (n = 4, *P < 0.05 by paired samples *t* test). **(B)** Cells from ALL6 or ALL17 were treated as described in Figure 2A. 4 h after IR, BCP-ALL blasts were harvested, lysed and subjected to immunoblot analysis with antibodies against p53 and actin or GAPDH.

The ability of both BM stroma and cAMP-enhancing agents to inhibit DNA damage-induced cell death led us to investigate whether stromal coculture would affect p53 levels. We cultured BCP-ALL cells from ALL5 with or without a supporting BM stromal colayer for 2 hours before subjecting the cells to 2 Gy of IR. After irradiation, BCP-ALL cells were allowed to remain in coculture for another 2 hours before being removed from the cocultures and analysed for the expression of p53. As can be seen from Figure 2A, unirradiated cells express low levels of p53. As expected, irradiation of the cells led to an approximately four-fold increase in p53 levels, an event that was significantly inhibited upon coculture of irradiated cells with BM stromal cells. Due to the relatively high numbers of cells required to perform SDS-PAGE/IB, we were not able to analyse the impact of stromal cells on p53 levels in all collected patient samples. However, stromal colayers were found to exert a similar inhibitory effect on p53 levels on cells from ALL6 and ALL17 as was shown for ALL5 (see Figure 2A and B). To exclude the possibility that the results obtained in Figures 1 and 2 could potentially be due to engulfment of early apoptotic cells by phagocytic cells present in the MSC colayer, thus selecting for more viable cells with less p53 available for analysis, we examined representative colayers with confocal microscopy and found no evidence of phagocytic activity by the iMSC cells (data not shown).

### MSC colayers produce PGE_2_

**Figure 3:**
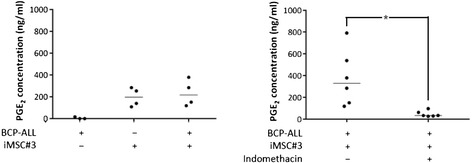
**iMSC#3 secretes PGE**_**2**_ **, which can be inhibited by Indomethacin.** iMSC#3 cells were grown to confluency, and growth medium was changed from MEMα to RPMI with or without Indomethacin as indicated. After 24 h, primary BCP-ALL cells were added as indicated and cocultures incubated for an additional 24 h. Supernatants were subsequently subjected to analysis of prostaglandin E_2_ levels by EIA as described in Materials and Methods. The results are presented as scatter plots with horizontal bars representing median values. *P < 0.05 by Wilcoxon matched pairs signed rank test.

Having demonstrated that colayers of MSC cells could mimic the effect of PGE_2_, cAMP induction, or PKA agonists on p53 levels and cell death in BCP-ALL cultures [11], we proceeded to examine the hypothesis that prosurvival signalling from BM stroma could in part be mediated through signalling cascades involving the PGE_2_-cAMP-PKA axis. To this end, we first wished to ensure that the stromal colayers indeed secreted PGE_2_. For this purpose iMSC#3 cells were grown to confluency before changing the growth medium. Supernatants were collected 24 hours after addition of fresh medium to the cell cultures, and PGE_2_ concentrations were measured as described in Materials and Methods. As can be seen from the left panel of Figure 3, the PGE_2_ concentrations in MSC supernatants varied between experiments, with a median value of approximately 200 ng/ml. The supernatants of BCP-ALL monocultures contained negligible levels of PGE_2_, indicating that autocrine supply of PGE_2_ by the leukaemic blasts is likely to be minimal. As there have been reports on paracrine loops in which cancer cells induce PGE_2_ synthesis in neighbouring MSCs or fibroblasts [23,27], we also measured the PGE_2_ concentration in supernatants of BCP-ALL/MSC cocultures. As can be seen from the left panel of Figure 3, there was no substantial induction of PGE_2_ synthesis by the MSC cells as a result of coculture. PGE_2_ is an eicosanoid produced from arachidonate by an enzymatic reaction catalysed by cyclooxygenase (COX), and the COX1/2 inhibitor indomethacin is a potent inhibitor of its biosynthesis. As expected, addition of indomethacin to the cocultures efficiently suppressed PGE_2_ production (Figure 3, right panel).

### The protective effect of MSC coculture depends on PGE_2_ production and PKA signalling

**Figure 4:**
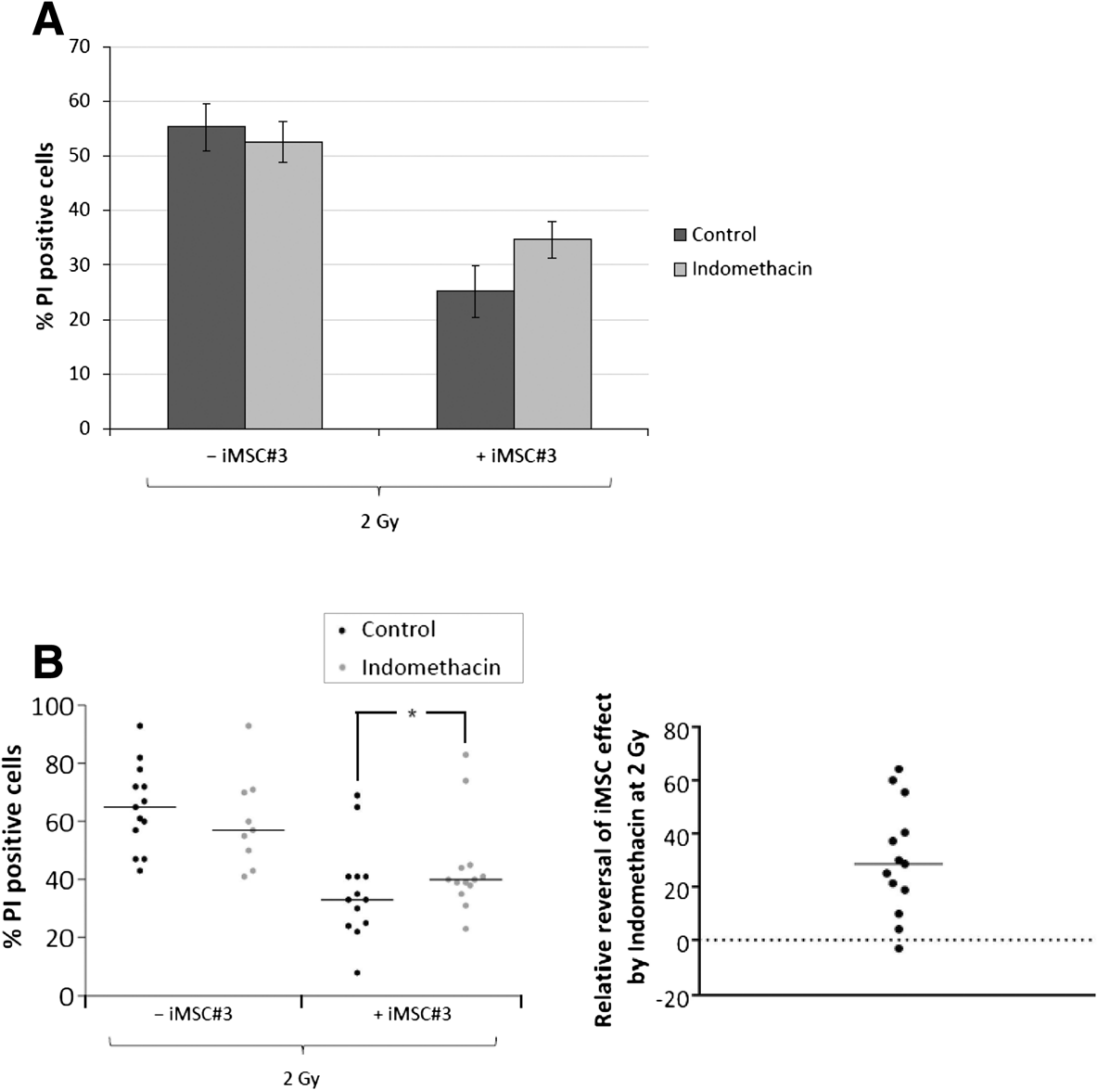
**Indomethacin reverses MSC-mediated cell death protection in BCP-ALL. (A)** iMSC#3 cells were grown to confluency before growth medium was changed from MEMα to RPMI with or without Indomethacin as indicated. After 24 h, ALL5 cells were cocultured with the iMSC#3 monolayer for 2 h before removal for irradiation with 2 Gy. BCP-ALL blasts were reintroduced to the iMSC#3 monolayer immediately after irradiation and cocultured for 20 h prior to cell death analysis of the CD19^+^ cell fraction by CD19-FITC/PI co-staining as described in Materials and Methods. The histogram shows mean values of five independent experiments with error bars indicating SEM values. **(B)** ALL blasts were cocultured, treated with Indomethacin, and analysed as described in 4A. Left panel: The scatter plot represents absolute cell death values from 13 independent experiments (ALL5: n = 5; ALL6: n = 1, ALL16: n = 4, ALL17: n = 1, ALL19: n = 1, ALL20: n = 1), with horizontal bars indicating median values. *P < 0.01 by Wilcoxon matched pairs signed rank test. Right panel: The relative reversal of iMSC#3 effect in irradiated (IR) cells by Indomethacin (Indo) was calculated according to the following formula: ([“+IR, +MSC, +Indo” − “+IR, +MSC, −Indo”] / [“+IR, −MSC, −Indo” − “+IR, +MSC, −Indo”]) × 100% . The values are displayed as a scatter plot with the horizontal bar representing the median value.

Having demonstrated that indomethacin inhibits the secretion of PGE_2_ by MSCs, we proceeded to investigate the possibility that indomethacin could reverse the prosurvival effect of iMSC#3 in cocultures. Monocultures or cocultures of BCP-ALL blasts from ALL5 were treated with 2 Gy of IR in the absence or presence of indomethacin. Whereas indomethacin had no effect on cell death of BCP-ALL blasts in monocultures, it reversed the protective effect of the MSC colayer by approximately 30% (Figure 4A). To test the reproducibility of this finding across samples from different patients, a series of experiments were performed on available t(12;21)-negative samples. t(12;21)-positive samples were excluded as these displayed a weak protective effect by MSC (see Figure 1C, lower panel). As shown in the left panel of Figure 4B, whereas indomethacin had a slight cytoprotective effect in the monoculture setting, it significantly reversed the prosurvival effect of MSC layeres in coculture. The relative indomethacin-induced reversal of the MSC-effect was calculated for each individual experiment, and as shown in the right panel of Figure 4B, the median reversal was 30% (range -3% to 64%). We also verified that indomethacin could reverse the protective effects of primary MSC layers upon cocultures with ALL 16 (data not shown).

**Figure 5:**
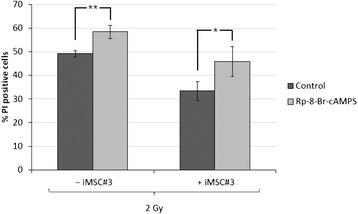
**PKA antagonist reverses MSC-mediated cell death protection in BCP-ALL.** iMSC#3 cells were grown to confluency before growth medium was changed from MEMα to RPMI with or without Rp-8-Br-cAMPS as indicated. After 24 h, ALL5 cells were cocultured with the iMSC#3 monolayer for 2 h before removal for irradiation with 2 Gy. BCP-ALL blasts were reintroduced to the iMSC#3 monolayer immediately after irradiation and cocultured for 20 h prior to cell death analysis of the CD19^+^ cell fraction by CD19-FITC/PI co-staining as described in Materials and Methods. The histogram shows mean values of seven independent experiments with error bars indicating SEM values. **P < 0.01, *P < 0.05 by paired samples *t* test.

To further investigate whether the prosurvival effects of MSC on BCP-ALL blasts could be conveyed by PGE_2_-induced cAMP-PKA signalling, we took advantage of the PKA antagonist Rp-8-Br-cAMPS. BCP-ALL blasts from ALL5 in monocultures or in cocultures with iMSC#3 were irradiated in the absence or presence of Rp-8-Br-cAMPS. Similar to indomethacin, Rp-8-Br-cAMPS significantly reversed the protective effect of MSC on IR-induced cell death in BCP-ALL blasts (Figure 5), indicating that PKA-mediated signalling is implicated in MSC-derived prosurvival signalling in ALL.

## Discussion

In the present paper we demonstrate that BM-derived MSCs in coculture protect primary BCP-ALL cells from DNA damage-induced p53 accumulation and cell death in a PGE_2_-dependent manner. To our knowledge, BM-induced suppression of p53 levels in leukaemia cells has not previously been reported. This novel finding may not only improve our understanding of how BCP-ALL develops, but also identifies the PGE_2_-cAMP-PKA signalling pathway as a possible target in the treatment of BCP-ALL.

**Figure 6:**
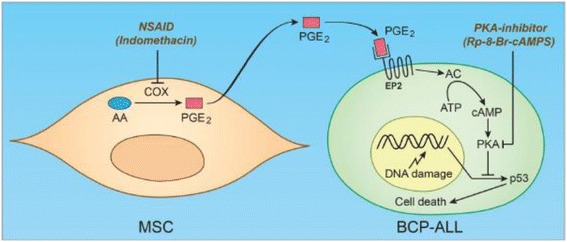
**Proposed model for MSC-derived PGE**_**2**_ **-mediated cytoprotection of BCP-ALL.** Mesenchymal stromal cells (MSC) produce prostaglandin E2 (PGE_2_) by the enzymatic conversion of arachidonic acid (AA) in a process depending on the action of cyclooxygenases (COX). PGE_2_ is then secreted into the extracellular space where it can signal in a paracrine manner by binding to the E prostanoid receptor 2 (EP2) on the surface of B cell precursor acute lymphoblastic leukaemia (BCP-ALL) cells. Binding of PGE_2_ to EP2 activates adenylate cyclase (AC) resulting in the enzymatic conversion of ATP to cAMP, which further activates target proteins such as protein kinase A (PKA). PKA activation attenuates p53 accumulation in BCP-ALL cells thereby inhibiting DNA damage-induced p53-dependent cell death. Non-steroidal anti-inflammatory drugs (NSAIDs) or PKA inhibitors can interfere with this cascade of events with the potential of reversing the MSC-mediated protection of BCP-ALL cells during DNA-damaging therapy.

Eicosanoids, and in particular PGE_2_, have an established role in cancer biology [26,28]. The role of PGE_2_ as an autocrine and paracrine signalling molecule has been particularly well-documented in carcinomas where it has been shown to promote tumour cell proliferation, survival, invasion and migration [26]. The anti-apoptotic activity of PGE_2_ in this setting has mainly been attributed to activation of the PI3K-Akt-PPARδ signalling axis [26]. To our knowledge, very little is known about the role of PGE_2_ in haematopoietic malignancies, and we here propose a distinct anti-apoptotic mechanism of PGE_2_ in BCP-ALLs compared to what is established in carcinomas. Based on our findings that 1) exposure of BCP-ALL cells to either forskolin, cAMP analogues, PGE_2_, or MSC cocultures inhibits p53 accumulation and cell death in a similar fashion [9-11], 2) MSCs secrete PGE_2_, 3) ALL cells are known to express functional EP2 receptors eliciting a cAMP response [24], and 4) the effect of MSC cocultures on BCP-ALL p53 levels and cell survival is inhibited by COX or PKA inhibitors, we suggest a model in which BCP-ALL cells are protected from DNA damage-induced p53 accumulation and cell death by BM stromal cells in a PGE_2_-cAMP-PKA-dependent manner (Figure 6). Furthermore, it should be noted that we have observed a similar effect of cAMP on p53 in carcinoma and sarcoma cell lines such as MCF-7 [10], U2-OS [10], and HCT116 (unpublished results), suggesting that inhibition of the tumour suppression function of wild type p53 by cAMP is not a phenomenon restricted to BCP-ALL.

A striking observation in the present study is the lack of cytoprotection of stromal coculture on blasts from t(12;21)-positive BCP-ALL patients (Figure 1C). This is in agreement with our previously reported lack of protective effect of forskolin on DNA damage-induced cell death in this cytogenetic subgroup of BCP-ALL [11], and further supports the possible contribution of cAMP-mediated signalling in MSC-driven prosurvival effects. Interestingly, t(12;21)-positive and -negative samples display similar p53 responses: p53 accumulates after irradiation, and the radiation-induced p53 response can be attenuated with the addition of forskolin or upon coculture with MSCs. However, in t(12;21)-positive samples the reduction in p53 does not translate into reduced levels of cell death [11]. One possible interpretation of this finding is that t(12;21)-positive cells have a lower threshold for p53-mediated induction of apoptosis than the other cytogenetic subgroups. The t(12;21) translocation results in the transcription of an ETV6/RUNX1 fusion protein and has been demonstrated to be the initiating event in the transformation of this subgroup of ALL, occurring *in utero* and resulting in a premalignant clone requiring additional secondary genetic alterations to develop into overt leukaemia [29-31]. It was recently published that the ETV6/RUNX1 fusion protein can bind to the P2 promoter of the *HDM2* gene and enhance its transcription [32]. This could result in an inherent protection of the t(12;21)-positive clones from oncogene-induced p53 accumulation. When challenged with severe DNA-damaging stress, such as ionising radiation or DNA-damaging cytostatic drugs, the elevated HDM2 levels in ETV6/RUNX1 positive BCP-ALL cells might no longer suffice to suppress p53 accumulation, and cells succumb to p53-induced apoptosis in accordance with our observations. In the presence of increased cAMP levels, for instance induced by stromal cocultures, p53 accumulates to a lesser extent, but appears to remain above a threshold for effective apoptosis induction. This might be in contrast to non-ETV6/RUNX1 expressing BCP-ALL cells, which would be expected to have evolved compensatory mechanisms to tolerate higher p53 levels.

Among the t(12;21)-negative BCP-ALLs there are cytogenetic subgroups with particularly poor prognosis, such as the t(9;22) (Philadelphia chromosome)-positive subtype represented by ALL5 in the present study [1]. Importantly, our findings suggest that pharmacological intervention of PGE_2_-cAMP-PKA-mediated signalling in t(12;21)-negative BCP-ALLs might provide a therapeutic possibility to counteract the unfavourable protective effects of the BM niche towards these cytogenetic subtypes of BCP-ALLs. An obvious pharmaceutical candidate based on our results is non-steroidal anti-inflammatory drugs (NSAIDs), of which indomethacin is an example. NSAIDs are well-established in clinical use as analgetic and anti-inflammatory drugs, and are tolerated with manageable side effects. PKA inhibitors such as Rp-8-Br-cAMPS are not yet approved for clinical use, but have been tested in animal models without apparent severe toxicity [33,34]. An alternative approach could be stimulation of opioid receptors, which belong to a subgroup of GPCRs coupled to G_αi_ subunits able to inhibit the AC and thereby cAMP generation. In line with the latter approach, it was recently published that D,L-methadone, through binding to opioid receptors, sensitized leukaemia cells to DNA damage-induced cell death by doxorubicin [35].

As previously discussed [11], the potential of inhibiting PGE_2_ synthesis might already be exploited in conventional BCP-ALL treatment. Glucocorticoids (GCs), which constitute a backbone of modern BCL-ALL treatment, are known to attenuate the synthesis of prostaglandins via inhibition of phospholipase A2 and COX2 expression [36]. The importance of GC-induced inhibition of PGE_2_ has to our knowledge mainly been discussed in relation to the anti-inflammatory properties of GCs, whereas more direct proapoptotic effects have been the focus of explaining its antileukaemic properties [37]. We have observed a reduction of PGE_2_ concentration in supernatants of MSCs treated with dexamethasone (data not shown). If a similar trend can be observed in clinical BM specimens collected before and after the onset of GC-based therapy, this would encourage further investigation into the role of PGE_2_ suppression in the antileukaemic effect of GCs. Complementing the understanding of the biological effects of GCs in leukaemia would be important, as GC resistance in BCP-ALL represents a major adverse prognostic factor, and the mechanisms of such resistance is incompletely understood [37,38].

## Conclusion

We believe our results presented in the present paper lend strong support to the hypothesis that BM-derived MSCs can supply BCP-ALL cells with prosurvival signals acting through a PGE_2_-cAMP-PKA-axis eventually attenuating the p53 response. This signalling cascade can be of importance both during the preleukaemic phase by suppressing oncogene-induced p53, and during leukaemia therapy by suppressing DNA damage-induced p53. Furthermore, our results suggest targets for pharmacological intervention in this signalling pathway to counteract the so called chemoprotective niche in the BM, either by inhibiting PGE_2_ production by NSAIDs or GCs, by inhibiting AC activation by stimulation of the opioid receptor, or by direct PKA inhibition. Given that our *in vitro* findings can be replicated in a clinical setting, we hope our results can ultimately contribute to improvement of current leukaemia treatment by allowing increased antileukaemic effect of a given dose of a cytostatic drug. This could be exploited either to increase the efficacy of current treatment regiments, or alternatively to reduce currently applied doses of cytostatic drugs to prevent side effects whilst retaining the antileukaemic efficacy.

## Methods

### Reagents, antibodies and radiation treatment

Forskolin and propidium iodide (PI) were purchased from Sigma-Aldrich (St. Louis, MO). Rp-8-Br-cAMPS was a kind gift from Professor Kjetil Taskén (The Biotechnology Center of Oslo, University of Oslo, Norway). Antibodies against p53 (DO-1) and actin (C-2) were from Santa Cruz Biotechnology (Santa Cruz, CA), whereas antibodies against GAPDH were from Sigma-Aldrich. C19-FITC antibodies were from Miltenyi Biotech (Bergisch Gladbach, Germany) and CD10-PE-Cy™5 from BD Pharmingen (San Jose, CA). For treatment with γ-radiation, cells were exposed to a ^137^Cs source at a dose rate of 4.3 Gy/min using a Gammacell irradiator from MSD Nordion (Ottawa, Canada).

### Cell lines, primary cell isolation and cell culture

The immortalised bone marrow derived human mesenchymal stroma cell line iMSC#3 [39] was generated by transduction with the pBabe-puro-hTert adeno virus [40], is nontumourigenic, has a normal karyotype, and has maintained its osteogenic and adipogenic potential, as well as the ability to support B cell maturation [41]. Cells were maintained at 30-80% confluence in MEM alpha medium supplemented with 10% foetal bovine serum (FBS), 100 U/ml penicillin, and 100 μg/ml streptomycin (PS). Cultures of primary bone marrow stromal cells were established from bone marrow samples obtained from healthy volunteers. 10 ml bone marrow aspirate was mixed with 500 μl 2% methylcellulose and left to sediment for 60 min. The buffy-coat was cultured at a density of 3×10^6^ cells/ml in IMDM medium supplemented with 12.5% horse serum, 12.5% FBS, 100 μM β-mercaptoethanol, 100 μM hydrocortisone, and PS. Adherent cells reached confluence after 2-3 weeks, and the cell culture was used for experiments within the first week thereafter. Alternatively, BM was diluted 1:3 in DMEM/F12-Glutamax before density centrifugation in Lymphoprep (Axis-Shield, Dundee, Scotland). Mononuclear cells were seeded, and adherent cells cultured as above. 24 hours prior to coculture with BCP-ALL blasts, the medium of MSC cells was changed to supplemented RPMI (see below). Primary BCP-ALL blasts were isolated from bone marrow aspirates performed at diagnosis as previously described [9]. The proportion of CD10^+^CD19^+^ blasts was assessed by costaining with CD10-PE-Cy™5 and CD19-FITC antibodies followed by flowcytometric analysis on a FACS Calibur instrument (BD Biosciences, NJ). BCP-ALL blasts were cultured at 10^6^ cells/ml in RPMI medium supplemented with 10% FBS and PS. The collection of bone marrow aspirates from patients and healthy volunteers has been approved by the Regional Ethics Committee of Norway, region Sør-Øst A, and recommended by Competent Authorities. Patient characteristics are outlined in Table 1.

**Table 1:** **Patient characteristics**

**Patient sample**	**ALL5**	**ALL6**	**ALL10**	**ALL12**	**ALL16**	**ALL17**	**ALL18**	**ALL19**	**ALL20**	**ALL 21**
Age (years.months)	14.5	5.0	15.6	3.4	2.8	3.2	6.5	7.5	7.4	11.7
Sex	Female	Female	Female	Female	Male	Male	Male	Male	Male	Male
Sample from relapse	No	Yes	No	No	No	No	No	No	No	No
Immunophenotype	Common-B	Common-B	Common-B	Pre-B	Pre-B	Pre-B	Pre-B	Pre-B	Pre-B	Pre-B
Cytogenetics	t(9;22)	Normal karyotype	t(12;21)t(5;17)	t(12;21)(p13;q22)	47 ~ 48,XY,+X,i(21)(q10),+ i(21)(q10),[cp9]/46,XY[1]	46 XY, - 21c	t(12,21)	44, X,-Y,t(4;14) (q25;q32	46,XY,t(1;19)(q(23;p13)[2]/46,XY[23]	t(12;21)(p13;q22)
Residual disease at day 15	42 × 10^-2^	Not performed	30 × 10^-2^	1.1 × 10^-2^	3.2 × 10^-3^	8.9 × 10^-3^	8 × 10^-3^	No MRD	5 x 10^-3^	No MRD
Residual disease at day 29	27 × 10^-2^	No MRD	7.3 × 10^-3^	3 × 10^-4^	No MRD	9 × 10^-4^	5 × 10^-4^	No MRD	No MRD	No MRD

MRD: minimal residual disease.

### Selection of patient samples for different experiments

Upon isolation of BCP-ALL blasts from patients, the resulting cell yield and viability after freezing and thawing was very variable between samples. Patient samples were prioritized into different experiments accordingly. Collection of samples was performed in continuity with previous projects [9,11], with samples ALL14-21 collected with primary use for the current project. ALL14 and 15 was excluded due to very poor viability and cell yield respectively. In addition, frozen material from previously collected material has been used when available (ALL5, 6, 10 and 12). As t(12;21)-positive samples display poor protection from MSC coculture, these were excluded from experiments examining the potential reversibility of this effect by COX or PKA inhibitors. Of available t(12;21)-negative samples, only cell yield/viability upon thawing from patient 5 and patient 16 was sufficient to perform repeated experiments with cocultures and COX or PKA inhibitors.

### Measurement of cell death, immunoblotting and densitometric analysis

Measurement of cell death in the CD19-positive fraction, immunoblot (IB) analysis, densitometric analysis, and normalization of densitometric values was performed as previously described [9,11].

### Quantification of PGE_2_ concentration in culture supernatant

The concentration of PGE_2_ in culture supernatants was determined by competitive enzyme immuno assay (EIA) using the Prostaglandin E_2_ EIA Kit – Monoclonal from Cayman Chemical Company (Ann Arbor, MI). Experiments were performed according to the manufacturer’s procedure. For each sample, two parallel aliquots were run, and the optical density of standards and samples at a wavelength of 405 nm was determined using a Multiscan Ex microplate reader (Thermo Scientific, Waltham, MA). The results were analysed using a data analysis tool provided online by the manufacturer at www.caymanchem.com/analysis/eia.

### Statistical methods and calculation

SPSS 14.0.2 for Windows was used to perform statistical analysis. The paired samples *t* test was used to test the significance of differences in series of experiments run on cells from the same patient source, whereas the Wilcoxon signed-rank test was used to test the significance of differences observed in series of experiments run on cells from different patients.

## Abbreviations

AC: Adenylate cyclase
BCP-ALL: B cell precursor acute lymphoblastic leukaemia
BM: Bone marrow
BMME: Bone marrow microenvironment
cAMP: Cyclic adenosine monophosphate
COX: Cyclooxygenase
EIA: Enzyme immuno assay
FBS: Foetal bovine serum
GC: Glucocorticoids
GPCR: G protein-coupled receptor
IB: Immunoblotting
IR: Ionising radiation
ME: Microenvironment
MSC: Mesenchymal stromal cell
NSAID: Non-steroidal anti-inflammatory drug
PGE_2_: Prostaglandin E_2_
PI: Propidium Iodide
PKA: Protein kinase A
PS: Penicillin-streptomycin
SDS-PAGE: Sodium dodecyl sulphate polyacrylamide gel electrophoresis
